# High-grade transformation of a polymorphous adenocarcinoma of the salivary gland: a case report and review of the literature

**DOI:** 10.3389/fonc.2023.1245043

**Published:** 2023-09-18

**Authors:** Giacomo Miserocchi, Massimo Bassi, Giovanni De Luca, Sebastiano Calpona, Francesco De Rosa, Alberto Bongiovanni, Elisabetta Parisi, Giandomenico Di Menna, Alessandro De Vita, Chiara Liverani, Chiara Spadazzi, Claudia Cocchi, Silvia Vanni, Laura Capelli, Massimo Magnani, Giuseppe Meccariello, Claudio Vicini, Angelo Campobassi, Laura Mercatali, Toni Ibrahim

**Affiliations:** ^1^ Preclinic and Osteoncology Unit, IRCCS Istituto Romagnolo per lo Studio dei Tumori (IRST) “Dino Amadori”, Meldola, Italy; ^2^ Maxillofacial Surgery Unit, “Bufalini Hospital”, Azienda Unità Sanitaria Locale (AUSL) Romagna, Cesena, Italy; ^3^ Pathology Unit, “Bufalini” Hospital, Azienda Unità Sanitaria Locale (AUSL) Romagna, Cesena, Italy; ^4^ Immunotherapy, Cell Therapy and Biobank, IRCCS Istituto Romagnolo per lo Studio dei Tumori (IRST) “Dino Amadori”, Meldola, Italy; ^5^ Radiotherapy Department, Istituto Scientifico Romagnolo per lo Studio e la Cura dei Tumori (IRST), IRCCS, Meldola, Italy; ^6^ Department of Medical Oncology, IRCCS Istituto Romagnolo per lo Studio dei Tumori (IRST) “Dino Amadori”, Meldola, Italy; ^7^ Biosciences Laboratory, IRCCS Istituto Romagnolo per lo Studio dei Tumori (IRST) “Dino Amadori”, Meldola, Italy; ^8^ Department of Head-Neck Surgery Azienda Unità Sanitaria Locale (AUSL) Romagna, Ear Nose Throat (ENT) Unit, Bufalini Hospital, Cesena, Italy; ^9^ Otolaryngology and Head-Neck Surgery Unit, Department of Head-Neck Surgeries, Morgagni Pierantoni Hospital, Azienda USL della Romagna, Forlì, Italy

**Keywords:** polymorphous adenocarcinoma, high-grade transformation, minor salivary gland, gene expression profiling, oral floor

## Abstract

**Background:**

Polymorphous adenocarcinoma (PAC) represents the second most widespread neoplasm of the minor salivary glands. These tumors rarely develop a histological progression from low-grade to high-grade malignancy, named “high-grade transformation” (HGT). Only nine cases are described in literature.

**Case description:**

Here, we describe the case of a 76-year-old male patient with a PAC recurrence of the oral floor displaying HGT, and we explore the tumor cytomorphological features, genomic profiling, and the patient’s clinical management. The tumor mass was characterized by poorly atypical cellular elements with vesicular nuclei and comedonecrosis foci. The growth pattern was predominantly solid, tubular, and cribriform. The lesion did not show microsatellite instability or targeted molecular alterations. The case was successfully treated with radical surgery followed by radiotherapy.

**Conclusion:**

We report for the first time the recurrence of a PAC with HGT arising in the oral floor after 20 years from the primary lesion. These preliminary data and the literature analysis enhance the knowledge of this extremely rare disease.

## 1 Introduction

Polymorphous adenocarcinoma (PAC) is the second most common malignancy of the minor salivary glands (1, 2). The incidence of salivary cancers is estimated to be 4–135 cases per million population per year and approximately 10%–15% are located in the minor salivary glands (3). PACs normally arise as surface papillary epithelial hyperplasia with stippled mucosa covering the cancer mass (4).

These tumors are described as infiltrative epithelial malignancies, showing bland nuclei, poor to moderate cytoplasm, and a variety of cytoarchitectural patterns, including solid, cribriform, tubules, and Indian-file infiltrates (1, 5–7). In the past decades, it is likely than PACs have been misdiagnosed as adenoid cystic carcinomas (AdCCs) (8). From a pathological point of view, the two salivary gland tumors display different cribriform patterns; whereas AdCC presents stromal cores surrounded by tumor parenchyma and thus characterized by basement membrane, PAC exhibits genuine lumina and a variety of myxoid, fibrous, hyalinized, or elastotic stroma with inconspicuous inflammation (1). Moreover, neurotropisms, as targetoid pattern, and perivascular arrangements are often detected. The latest WHO categorization of salivary gland tumors includes the so-called “cribriform adenocarcinoma of minor salivary glands” (CAMSG) under the PAC heading, despite their important differences in clinical behavior (7). Unlike PAC, CAMSG usually arises in the base of the tongue and shows more aggressive clinical behaviors, such as higher risk of lymph node metastasis (7). Moreover, CAMSG tumor cells are characterized by vesicular and pale nuclei with ground-glass appearance and clear to eosinophilic cytoplasm (2).

PACs were initially described as indolent malignancies with low metastatic potential (9). However, recent clinical evidence has reported recurrences in 19% of PACs and extremely rare cases of high-grade transformation (HGT) (10–12), with development of cytological atypia, increased proliferative activity, and necrosis areas (11).

In the literature, nine cases of PAC displaying high-grade features have been described (11–17). These pathological conditions are heterogeneous in terms of clinical outcomes, metastasis onset, and treatments; can arise in both primary tumors and recurrences; and can be located in several sites: palate, nasal cavity, maxillary alveolus, and upper lip.

In this study, we review the state- of- the-art literature on this extremely rare salivary gland cancer, and report the clinical and genomic characterization of a new case of PAC characterized by both high- and low-grade aspects.

## 2 Case presentation

### 2.1 Case report

In 2000, a 54- year-old male patient underwent a left neck node dissection of levels 2–5 and multiple biopsies (larynx, base of the tongue, amygdaloglossus sulcus, and tonsil) for a metastatic node with no clinically evident primary lesion. The histology confirmed a malignant lymphadenopathy with the presence of a single lymph node metastasis with extranodal lymphatic vascular infiltration. The primitive lesion was not detected. The metastatic tissue showed a tubular, follicular, and microcystic glandular structure with hyaline stroma and crystalloid and amorphous material deposition. The sample displayed necrotic foci, while cancer cells showed poor nuclear atypia and irrelevant mitotic activity. Immunohistochemical analysis revealed α-SMA positivity in peripheral spindle cells of tubular structures and S100 focal positivity. Thyroglobulin, calcitonin, and chromogranin staining were negative. The other lymph nodes appeared with reactive and aspecific modifications. Therefore, the pathologic diagnosis identified a cribriform variant of a lymph node metastasis with low-grade histologic features and probably originated from a primitive neoplasia of the salivary gland, pathologically staged as TX N1 M0. No adjuvant chemotherapy or other treatments were performed due to the low-grade nature of the lesion.

**Figure 1:**
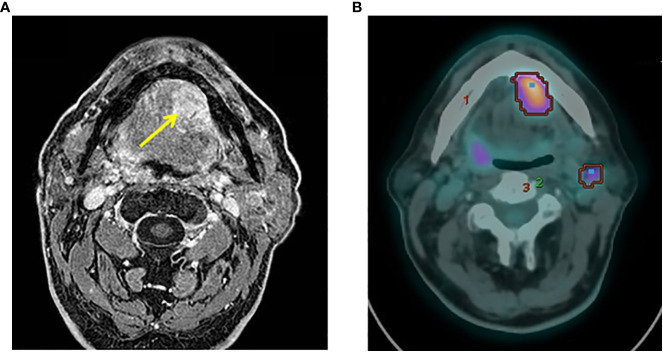
Preoperative NMR and PET examinations. **(A)** Axial view of preoperative NMR scan showing the tumor mass (yellow arrow). **(B)** PET images revealed a focal hyperfixation area of the left oral floor and two concomitant areas of radioisotope uptake in the left submandibular and unilateral mandibular seat of the lymph nodes.

In January 2020, the patient referred to the Oral and Maxillofacial Surgery Unit of Cesena “Bufalini Hospital” for a swollen nodule in the left of the oral floor. Magnetic resonance imaging (MRI), using T1-weighted high-resolution isotropic volume examination (THRIVE), confirmed the presence of a single lesion in the left portion of the oral floor involving the unilateral sublingual space (**Figure 1A**). The mass was well delimited and in contact with the mandibular cortex, with no signs of bone infiltration: diameters of 2.4 × 3 cm in the axial sequence, 2 × 2.8 cm in the coronal sequence, and 3. cm in the sagittal sequence. In the loco-regional seat, the bone cortex of the jaw appeared remodeled and thin but not interrupted and with no alteration signal, while reaching the ventral surface of the lingual root and the mylohyoid muscle. A biopsy was subsequently performed. On the back, the lesion was widespread since the ventral surface of the tongue root. The mass reached the left mylohyoid muscle with the removal of adipocytic cleavage floor. However, the lesion did not appear to have passed the muscle and was not extended to the submandibular space. The left submandibular gland was characterized by a poor oversized extra- and intraglandular ductal system. The Wharton duct appeared dilated as probably due to the obstruction/infiltration of the excretory duct by the cancer lesion. The volume of the left parotid gland was poorly increased as compared to its counterpart and showed two formations visible in the deep lobe: one of 10 mm characterized by mixed signal (hypo- and hyper-intense) and the other of 14 mm with hemorrhagic component. An additional formation of 15 mm in the inferior pole of the parotid gland (level IIA) and a neoformation in the left oral floor were revealed.

**Figure 2:**
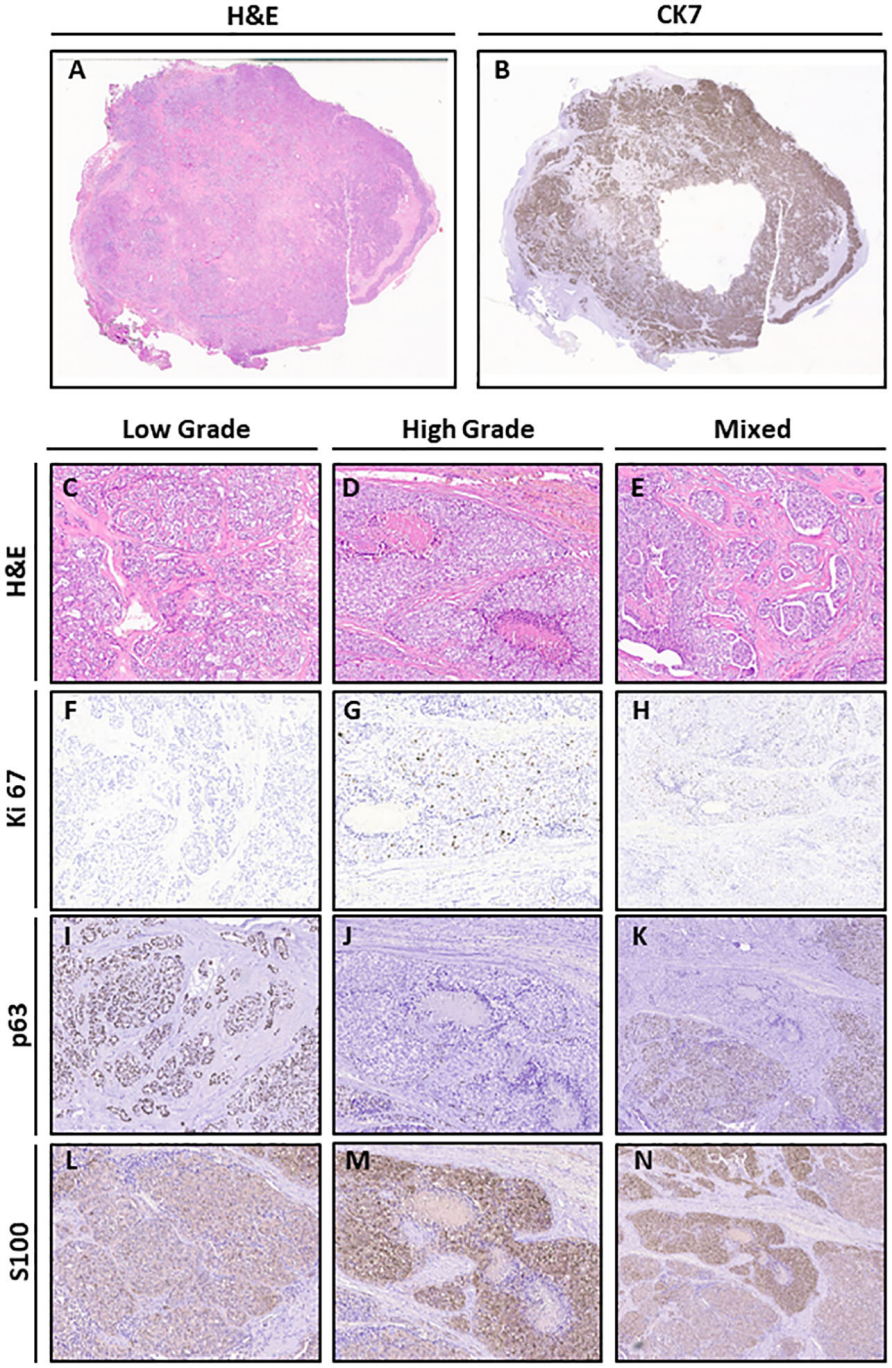
Histological features of the lesion. **(A)** H&E (original magnification 1×). **(B)** CK7 (original magnification 1×). **(C–E)** H&E of Low Grade, High Grade (original magnification 10×), and Mixed (original magnification 4×) areas. **(F–H)** Ki67 staining of Low Grade, High Grade (original magnification 10×), and Mixed (original magnification 4×) areas (H&E original magnification 10×). **(I–K)** p63 staining of Low Grade, High Grade (original magnification 10×), and Mixed (original magnification 4×) areas. **(L–N)** S100 staining of Low Grade, High Grade (original magnification 10×), and Mixed (original magnification 4×) areas.

In February 2020, a biopsy was performed and the pathology report described a glandular parenchyma partially substituted by malignant components with cribriform aspects. Tumor cells appeared monomorphic, without significant atypia, with nuclei of medium dimension, vesicular and no evident nucleolus. The H&E staining revealed the presence of low- and high-grade tumor features (**Figures 2A, C–E**), with different biomarker expression on immunohistochemistry assays. The whole tumor tissue was positive for cytokeratin 7 (**Figure 2B**), while Ki67 staining showed a lower cell proliferation in the low-grade areas with respect to the high-grade counterparts (**Figures 2F–H**). Cells of the low-grade areas showed higher expression of p63 and lower expression of S100 (**Figures 2I–N**). The whole tumor areas were negative for p40 (**Supplementary Figures 1A, B**). Owing to the histological features and to the tumor site, the case was diagnosed as a cribriform variant of a salivary gland PAC and considered as a recurrence with HGT of the metastatic lesion of unknown primary resected in 2000, the lesion being a cribriform variant of a salivary gland PAC. The tumor was classified as a recurrence of the unknown primitive of 2000 due to the very similar histological features.

In March 2020, ^18^F- fluorodeoxyglucose (^18^F-FDG) positron emission tomography/computed tomography (PET/CT) images revealed a focal uptake of the left oral floor with a maximum standardized uptake value (SUV) of 9. Two concomitant areas of radiotracer uptake were also present in the left submandibular (SUV max = 4.9) and ipsilateral mandibular (SUV max = 3.8) lymph nodes (**Figure 1B**). No other uptake areas were detected. Therefore, the MTB (ENT and maxillofacial surgeons, pathologist, radiologist, medical, and radiation oncologists) opted for radical surgery.

**Figure 3:**
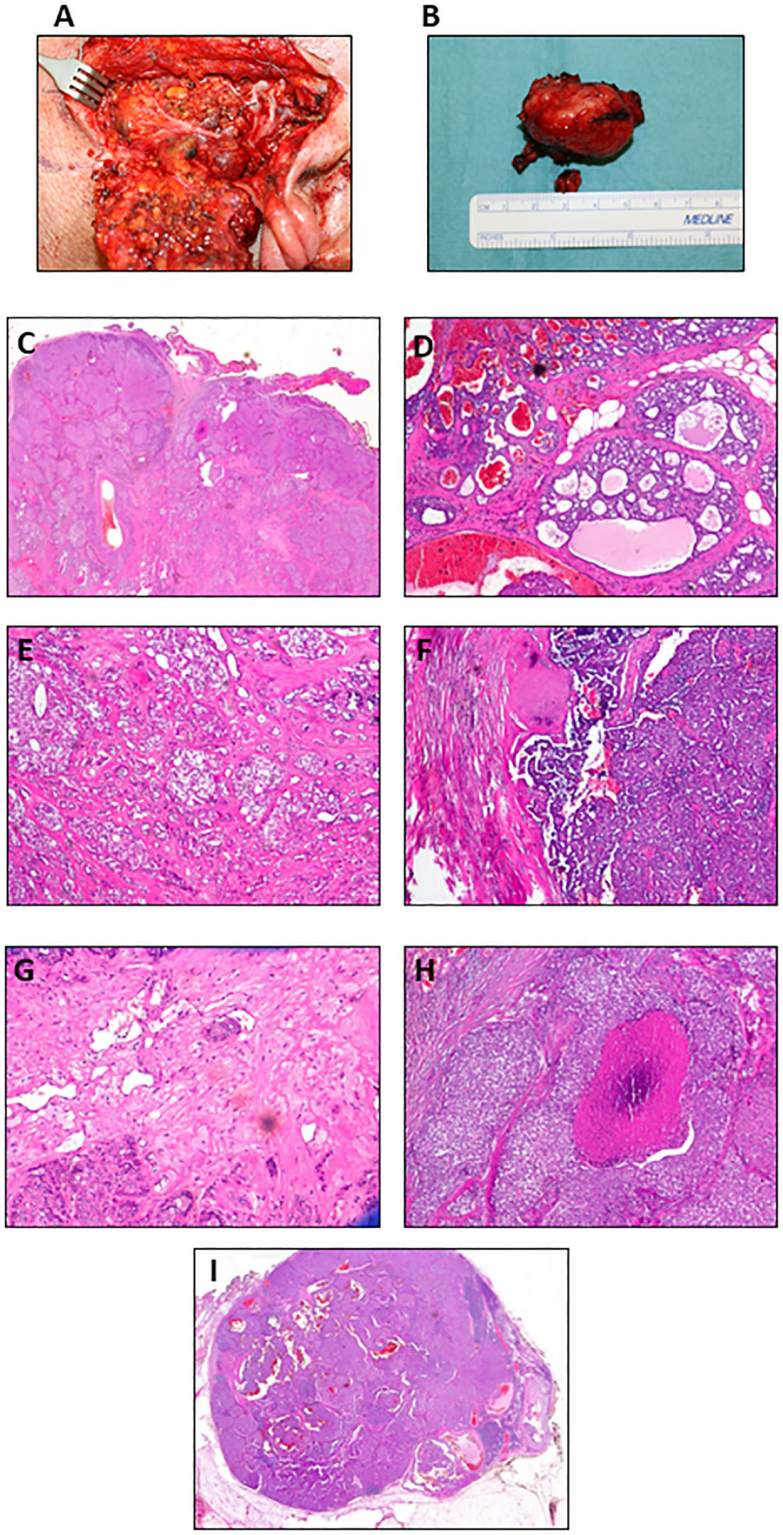
Postoperative images of the tumor mass and histological features. **(A)** Intraoperative photograph. **(B)** Lesion surgically excised. H&E images show **(C)** solid (original magnification 5×), **(D)** cribriform with focal infiltration in the perilesional adipose tissue (original magnification 10×), **(E)** cribriform and glandular (original magnification 10×), and **(F)** papillary growth pattern (original magnification 10×). Representative histopathological features: **(G)** vascular neoplastic invasion (original magnification 20×), **(H)** comedonecrosis (original magnification 10×), and **(I)** lymph node metastasis (original magnification 5×).

In May 2020, the patient underwent complete parotidectomy and dissection of the level 1 lymph node (**Figure 3A**). The surgery did not require microvascular or local flap reconstruction and no complications arose, allowing patient discharge after 7 days. The pathology report described a well-delimited solid mass (3.5 × 2.5 × 2 cm, weighing 15 g) (**Figure 3B**), not capsulated and showing marginal foci with infiltrative aspects. The tumor cells were atypical, with vesicular nuclei and evident nucleoli. The growth pattern was mostly solid (**Figure 3C**), with areas characterized by tubular and cribriform patterns (**Figures 3D, E**), as well as cystic areas with luminal papillary projections (**Figure 3F**). Vascular and perineural invasion were present (**Figure 3G**), together with multiple comedonecrosis foci (**Figure 3H**). Three lymph node metastases were detected. The degrees of lymph node involvement and growth patterns were variable (solid, cribriform, tubular, papillary, and cystic), but no aspects of extension to perilymphatic soft tissues were detected. The biggest measured was 1.4 cm (**Figure 3I**); therefore, the disease was staged as pT2, N2b according to TNM classification (18). The patient had no complications after surgery and he was discharged after 7 days.

Because of the presence of multiple lymph node metastases, the MTB referred the patient to adjuvant radiotherapy, completed in August (60 Gy/30 fractions to the tumor bed and ipsilateral nodes, 54 Gy/30 fractions to contralateral nodes). The treatment caused the onset of odynophagia, oral mucositis, and epitheliolysis in the left base of the neck, treated with supportive care and quickly resolved; the single long-term sequela was mild xerostomia.

In May 2023, the patient was still alive and MRI (**Supplementary Figure 2**) and PET/CT showed no sign of recurrence.

### 2.2 Molecular characterization

To the best of our knowledge, only one case of PAC with HGT has been molecularly characterized. Here, we performed NGS profiling and microsatellite instability (MSI) status using DNA and RNA extracted by tumor tissue. Sequencing analysis did not detect any alteration in the 52 genes of the NGS panel employed (**Supplementary Table 1**; **Data Sheet 1**); MSI status was obtained through the investigation of eight markers (**Supplementary Table 2** and **Data Sheet 2**). The analysis revealed an overlapping stable trend of the curves showed a ΔTm melting temperature (Tm sample − Tm positive control) ≥ −3 (unstable markers are considered for a ΔTm < −3) (**Supplementary Figure 3**). These results demonstrated the microsatellite stability of the sample.

## 3 Discussion

PAC is a rare malignancy of the minor salivary glands (1, 2), which usually arises in the palate (approximately 60% of all cases), lip, buccal mucosa, alveolar ridge, retromolar region, mouth floor, posterior tongue, and nasal cavity (1, 5, 6, 10, 12). Their nature is generally indolent and clinical outcome is positive, with local recurrence observed in 10%–30% of cases and regional metastases in approximately 15% (19). Histologically, PACs are described as malignant epithelial cancers characterized by heterogenic morphology, cytological uniformity, and an infiltrative growth pattern (7). The last WHO classification also includes CAMSG in the group of PAC variants (7). Different groups consider this aspect controversial and proposed to classify CASG as distinct lesions separated from PACs. Indeed, the two entities show differential diagnosis based on the patient’s history and histological examination. PACs are characterized by a heterogeneous group of growth pattern, including cribriform structures, the presence of concentric whorls developed by streaming columns of a single file or narrow trabeculae, and invasion of surrounding tissues and perineural spaces (20). Differently, CAMSGs were cytologically monomorphous with a limited range of growth patterns with a predominance of solid and cribriform structures mixed with a tubular pattern, mild cellular atypia, lymphovascular invasion, and infiltration of adjacent tissues (20, 21). Despite the invasive growth pattern of the two kinds of tumor, the overall prognosis remains favorable. Based on these differences, we have diagnosed the lesion here presented as PAC.

Originally identified as polymorphous low-grade adenocarcinomas (PLGAs), the WHO classification has changed the name in PAC, owing to the occurrence of sporadic cases characterized by a more aggressive pathophysiological feature and morphological appearance (7). These events are extremely rare and entail the progression from a low to high grade. High-grade PAC are characterized by prominent nucleoli, nuclear atypia, a high mitotic count, frequent central hemorrhage, and necrosis (1, 15, 22). Based on the histologic features, cytology, and behavior differences between PACs and CAMSGs, we have diagnosed the lesion here presented as PAC.

In the literature, the first documented case of PAC of the palate showing HGT at relapse was published in 1984 (13): four other cases subsequently reported features of HGT in PAC recurrences (11, 15). Pelkey et al. described two multiple locoregional recurrences displaying histological transformation to high grade after 17 and 26 years (15). The fourth and fifth cases recurred after 11 and 28 years, respectively (11, 17). Here, we report the first documented case of PAC recurrence that occurred 20 years after a lymph node metastasis of unknown primary lesion with low-grade histologic aspects. Although late recurrences were already described, the case here reported shows unusual and unique features not only in the recurrence, but also in the primary tumor. Indeed, the diagnosis of the second lesion as a recurrence with HGT of the undetected primary tumor relies on the same histologic features shared with the lymph node metastasis of 2000. The supposedly spontaneous remission of the primary PAC confirms the low aggressiveness of the disease.

In some instances, high-grade morphological features were also identified in PAC at initial presentation (11, 12, 14, 16). The tumor recurrences arose only in the palate while the cases at the initial presentation also included the site of nasopharynx and maxillary alveolus (14, 16). Therefore, our report describes the first documented case originating from the oral floor.

PAC and AdCC share many growth pattern features, such as solid and cribriform histology or the presence of neurotropism (6, 12): immunohistochemical stainings for the myoepithelial markers α-SMA and p40, positive in AdCC and negative in PAC, help to discriminate between the two entities (12, 23–25).

PACs with HGT —such as the case with our patient —share both high- and low-grade histological characteristics. In particular, high-grade areas are characterized by a solid growth pattern and necrosis or comedonecrosis foci, and low-grade areas show heterogeneous growth patterns (solid, tubular, trabecular, and cribriform are the most represented) (**Table 1**). Our case presented a prevalent solid growth pattern with some areas with cystic, cribriform, and tubular features.

**Table 1:** Patients’ characteristics.

Patient	Sex, age (at surgery)	Site	Metastasis	Primary lesion/Recurrence	Histological growth patterns	Outcome (months)	Ref.
1	F, 48	Palate	Lymph nodes, mandible	Recurrence	So, Cys, Pa	AWD (NA)	(13)
2	M, 47	Nasal cavity	NA	Primary lesion	So	Died for septic shock	(14)
3	F, 44	Palate	Lymph nodes	Recurrence	So, Crib, Tra, Sin-F	NA	(15)
4	F, 38	Palate	/	Recurrence	Crib, Tub, Tra, Sin-F	NA	(15)
5	M, 66	Palate	/	Recurrence	So, Pa, Crib, Sin-F, Tub	AWD (156)	(11)
6	M, 63	Palate	Lymph nodes	Primary lesion	So, Crib, Tub, Sin-F	AWD (5)	(11)
7	F, 73	Maxillary alveolous	Lymph nodes, abdomen, lung	Primary lesion	So, Crib, Tub, Sin-F	NA	(16)
8	M, 43	Palate	/	Primary lesion	So, Pa, Crib, Sin-F, Tub	AWD (39)	(12)
9	F, 73	Palate	/	Recurrence	So, Tra, Tub	NA	(17)
10	M, 74	Oral Floor	Lymph nodes	Recurrence	So, Cys, Pa, Crib, Tub	AWD (38)	

F, female; M, male; So, solid; Cys, Cystic; Pa, papillary; Crib, cribriform; Tra, trabecular; Sin-F, single-file (Indian-file); Tub, tubule; ADW, alive without disease; NA, not available.

“/” correspond to the absence of Metastasis.

As previously described, patients affected by PAC present good clinical outcomes, and this aspect is maintained also in the HGT variants. Indeed, four patients were alive and disease-free at the time of the case report publications (11–13) and only one had died from septic shock after an *Escherichia coli* infection of the urinary tract (14). In the other cases, the clinical outcomes were not available. In all cases, surgery represented the first treatment choice. In addition, radiotherapy was used in different modalities: alone (14), in combination with hyperthermia (13), or in combination with chemotherapy (15). In a single instance, the patient was treated with multidrug chemotherapy alone as adjuvant therapy after resection of multiple bilateral nodal metastases in the neck (11).

The mutational status of PACs with HGT are poorly described. Currently, only one study investigated the genome profiling of high-grade forms of PAC (17). The genomic analysis revealed a clonal NOTCH2 Q2409* truncating mutation and a MEF2B P315Qfs* frameshift mutation. Moreover, fluorescence *in situ* hybridization (FISH) analysis revealed PRKD2 rearrangement and PRKD1 and PRKD3 wild-type status. We used an NGS multi-biomarker assay to detect variants across cancer-relevant genes from DNA and RNA. No gene mutations were detected, neither in DNA nor in RNA. These results add genomic information on this extremely rare type of cancer, but further and more extended sequencing analysis are warranted to better characterize these diseases.

For the first time here, we explored the MSI status of a PAC with HGT. MSI represents a genetic hypermutability condition driven by DNA mismatch repair system (MMR) mainly associated with endometrial and gastric malignancies (26–28). MSI analysis showed an overlapping stable trend of the curves, and thus, microsatellites were considered stable. Several studies associate higher MSI frequency to young non-smoker patients with H&N SCCs (29, 30) while salivary gland tumors display lower frequency (31). Therefore, the case presented here is consistent with the scientific literature and suggests that HGT does not influence the stability of microsatellites. However, further analyses on different tumor samples are needed to better describe the genetic status of this rare disease.

Taken together, our results and the scientific evidence on these extremely rare malignancies highlight some considerations. HGT in salivary gland carcinomas is a process associated with a more aggressive behavior and poorer prognosis with respect to low-grade forms (22, 32). Conversely, the cases of PAC with HGT described in literature and summarized in this review (**Table 1**) showed heterogeneous clinical outcomes. Indeed, only in one case did the patient die as a consequence of his tumor (14). These differences suggest that HGT of PAC might be associated with a less aggressive behavior compared to the other salivary gland carcinomas.

## Data availability statement

The datasets presented in this article are not readily available because of ethical/privacy restrictions. Requests to access the datasets should be directed to the corresponding author.

## Ethics statement

The studies involving humans were approved by IRST-Area Vasta Romagna Ethics Committee. The studies were conducted in accordance with the local legislation and institutional requirements. The participants provided their written informed consent to participate in this study. Written informed consent was obtained from the individual(s) for the publication of any potentially identifiable images or data included in this article.

## Author contributions

Conceptualization, GMi, MB, GDL, LM, and TI; methodology, GMi, MB, GDL, SC, FDR, AB, EP, GDM, MM, GMe, CV, and AC; formal analysis, GMi, MB, GDL, SC, FDR, AB, and EP; investigation, GMi, MB, GDL, EP, GDM, ADV, CL, MM, GMe, CV, and AC; data curation, GMi, MB, GDL, ADV, CL, CS, CC, SV, and LC; writing—original draft preparation, GMi, ADV, CL, CS, CC, SV, and LC; writing—review and editing, GMi, MB, GDL, SC, FDR, AB, EP, GDM, MM, GMe, CV, AC, LM, and TI; supervision, LM and TI. All authors contributed to the article and approved the submitted version.
