# Supplementary material for: High-grade transformation of a polymorphous adenocarcinoma of the salivary gland: a case report and review of the literature
Source: Front Oncol. 2023 Sep 18;13:1245043. doi: 10.3389/fonc.2023.1245043 (PMC10545860; doi:10.3389/fonc.2023.1245043)
Supplement: Supplementary file 1 [file DataSheet_1.docx]

Supplementary Material

High-grade transformation of a polymorphous low-grade adenocarcinoma of the salivary gland: a case report and review of the literature

**Giacomo Miserocchi*, Massimo Bassi, Giovanni De Luca, Sebastiano Calpona, Francesco De Rosa, Alberto Bongiovanni, Elisabetta Parisi, Giandomenico Di Menna, Alessandro De Vita, Chiara Liverani, Chiara Spadazzi, Claudia Cocchi, Silvia Vanni, Laura Capelli, Massimo Magnani, Giuseppe Meccariello, Claudio Vicini, Angelo Campobassi, Laura Mercatali and Toni Ibrahim**

*** Correspondence:** Corresponding Author: [giacomo.miserocchi@irst.emr.it](mailto:giacomo.miserocchi@irst.emr.it)

**Supplementary Table 1.** List of hotspot mutations, CNV targets and gene fusions analyzed.

| Hotspot genes | AKT1, ALK, AR, BRAF, CDK4, CTNNB1, DDR2, EGFR, ERBB2, ERBB3, ERBB4, ESR1, FGFR2, FGFR3, GNA11, GNAQ, HRAS, IDH1, IDH2, JAK1, JAK2, JAK3, KIT, KRAS, MAP2K1, MAP2K2, MET, MTOR, NRAS, PDGFRA, PIK3CA, RAF1, RET, ROS1, SMO |
| --- | --- |
| Copy number genes | ALK, AR, BRAF, CCND1, CDK4, CDK6, EGFR, ERBB2, FGFR1, FGFR2, FGFR3, FGFR4, KIT, KRAS, MET, MYC, MYCN, PDGFRA, PIK3CA |
| Gene fusions | ABL1, AKT3, ALK, AXL, BRAF, EGFR, ERBB2, ERG, ETV1, ETV4, ETV5, FGFR1, FGFR2, FGFR3, MET, NTRK1, NTRK2, NTRK3, PDGFRA, PPARG, RAF1, RET, ROS1 |

**Supplementary Data Sheet 1**

*Next-generation sequencing assay and data analysis*

DNA and RNA were obtained from FFPE sections. MagMAX FFPE DNA/RNA Ultra Kit (Thermo Fisher Scientific) was used to extract the nucleic acids, following manufacturer’s protocol.

Next-generation sequencing (NGS) assay was carried out on DNA and RNA extracted from patients’ tissue. Qubit 2.0 Fluorimeter (Thermo Fisher Scientific) was used to determine DNA and RNA sample concentrations following the manufacturer’s protocol. 10ng of RNA were employed to perform the complementary DNA (cDNA) synthesis using SuperScript™ VILO™ cDNA Synthesis Kit (Thermo Fisher Scientific). Libraries and template preparation were performed with Ion Chef System (Thermo Fisher Scientific) using the DNA and cDNA samples. 12 ng of DNA was prepared as input with the Oncomine Focus Assay, Chef-Ready Library Kit. Ion 510™ & Ion 520™ & Ion 530™ Kit – Chef was used for template preparation. The sequencing assay was performed using the Ion GeneStudio™ S5 Plus System (Thermo Fisher Scientific). Hotspot mutations, copy number variations (CNV) targets and gene fusions were analyzed for all the genes reported in the Supplementary Table 1.

Data analysis was performed using Ion Torrent Suite™ Browser version 5.12. Sequence alignment and the detection of CNVs, SNVs, 5'-to-3' imbalance and specific gene fusions were carried out uploading the unaligned binary files (uBAM) in the Ion Reporter™ Software (IR) 5.10 (ThermoFisher Scientific). A custom filter chain has been set with a cut off 500X coverage and a minimum VAF 5% of alterations to identify gene variants. Fusions were detected considering a minimum number of >20 reads. The Coverage Analysis plugin was used to assess amplicon coverage for regions of interest and applied to all data.

**Supplementary Table 2.** complete list of markers and genes analyzed to determinate the MSI status.

| **Genes** | **Markers** | **NCBI Ref. Seq. (GRCh 38.p12)** |
| --- | --- | --- |
| cKIT | BAT25 | NC_000004.12 |
| MSH2 | BAT26 | NC_000002.12 |
| SLC7A8 | NR21 | NC_000014.9 |
| STT3A | NR22 | NC_000011.10 |
| ZNF2 | NR24 | NC_000002.12 |
| BIRC3 | NR27 | NC_000011.10 |
| CASP2 | CAT25 | NC_000007.14 |
| MAP4K3 | MONO27 | NC_000002.12 |

**Supplementary Data Sheet 2**

*Microsatellite instability (MSI) status evaluation*

Tumor DNA was employed to evaluate the microsatellite instability status using the EasyPGX ready MSI kit (Diatech Pharmacogenetics). Eight target gene were analyzed for (MSI) status (the complete list of markers and genes is summarized in Supplementary Table 2). The EasyPGX ® Analysis Software was used for data analysis comparing our results with a control sample included in the run.


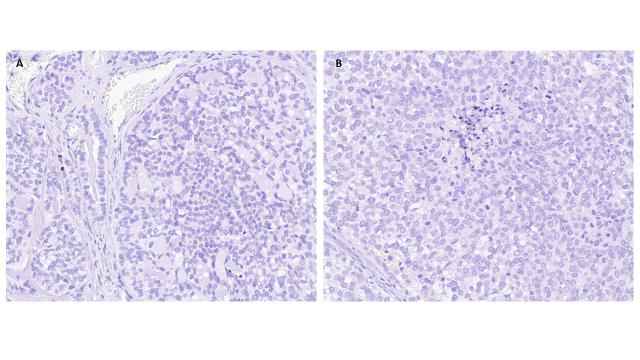


**Supplementary Figure 1,** p40 staining of the tumor tissue. (**A)** p40 staining of Low Grade area (original magnification 20 X) (**B**) and of High Grade area (original magnification 20 X).

**
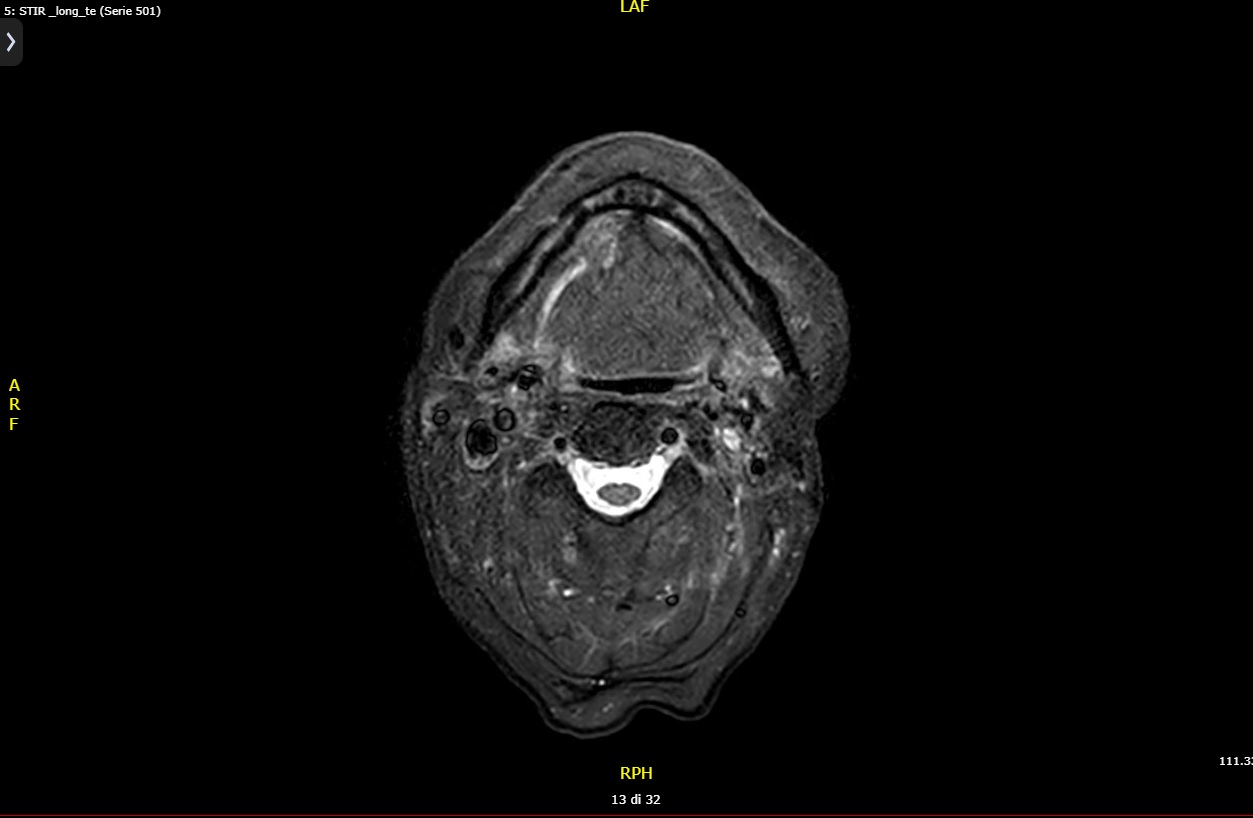
**

**Supplementary Figure 2** Postoperative NMR examinations. (**A)** Axial view of postoperative NMR scan showing the absent of malignant lesions.


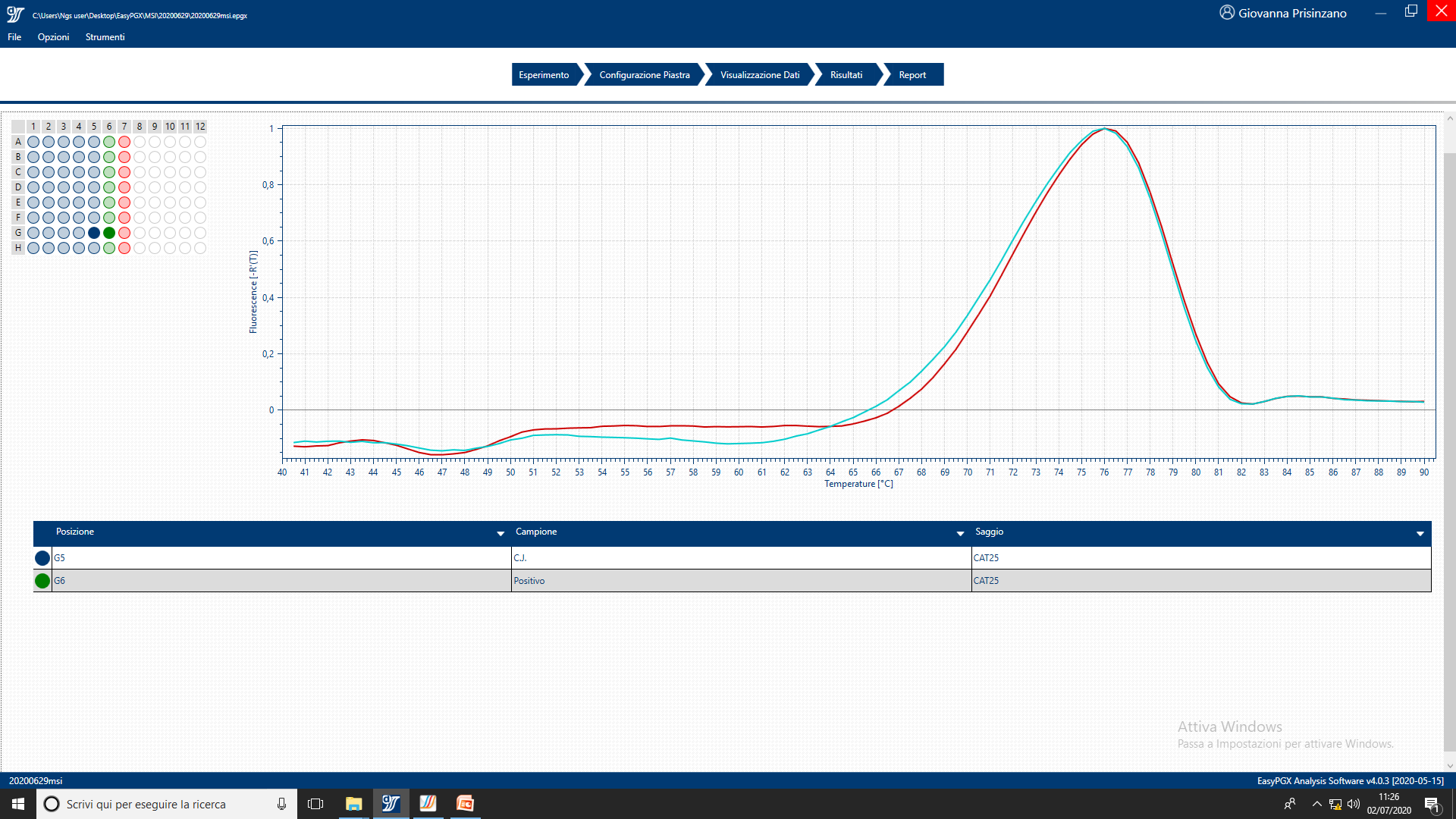

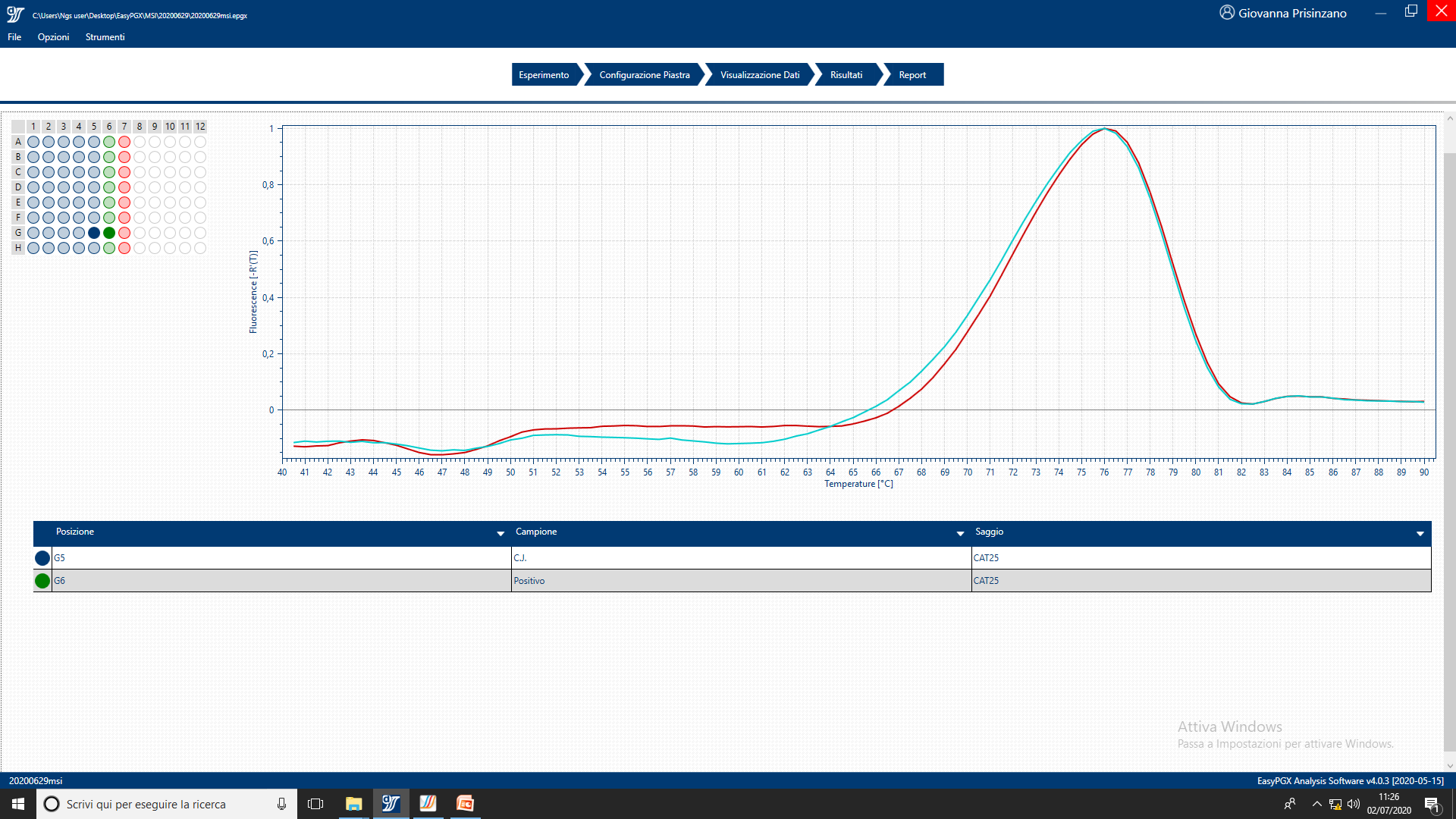


**PASG1**

**C-**


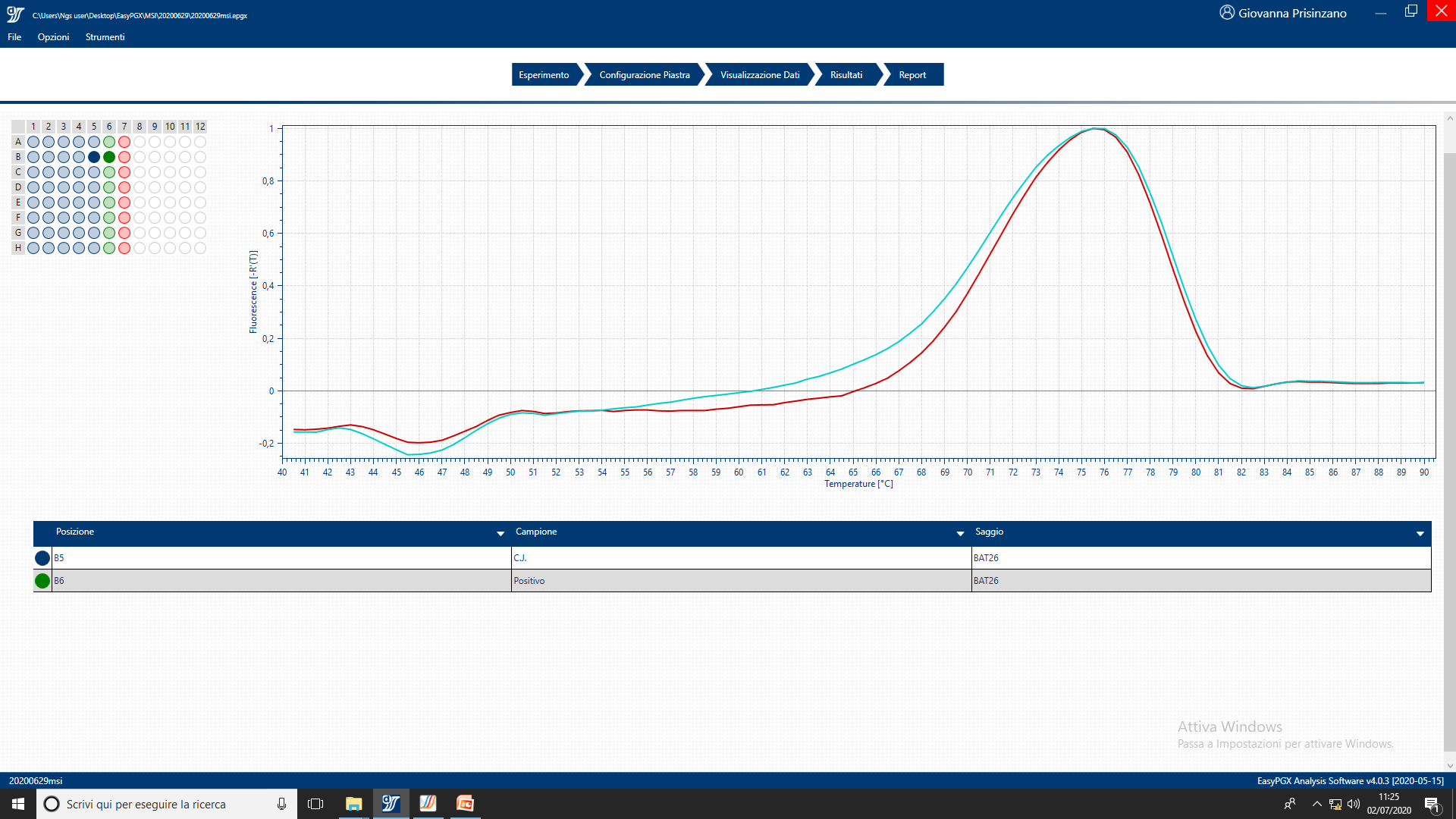

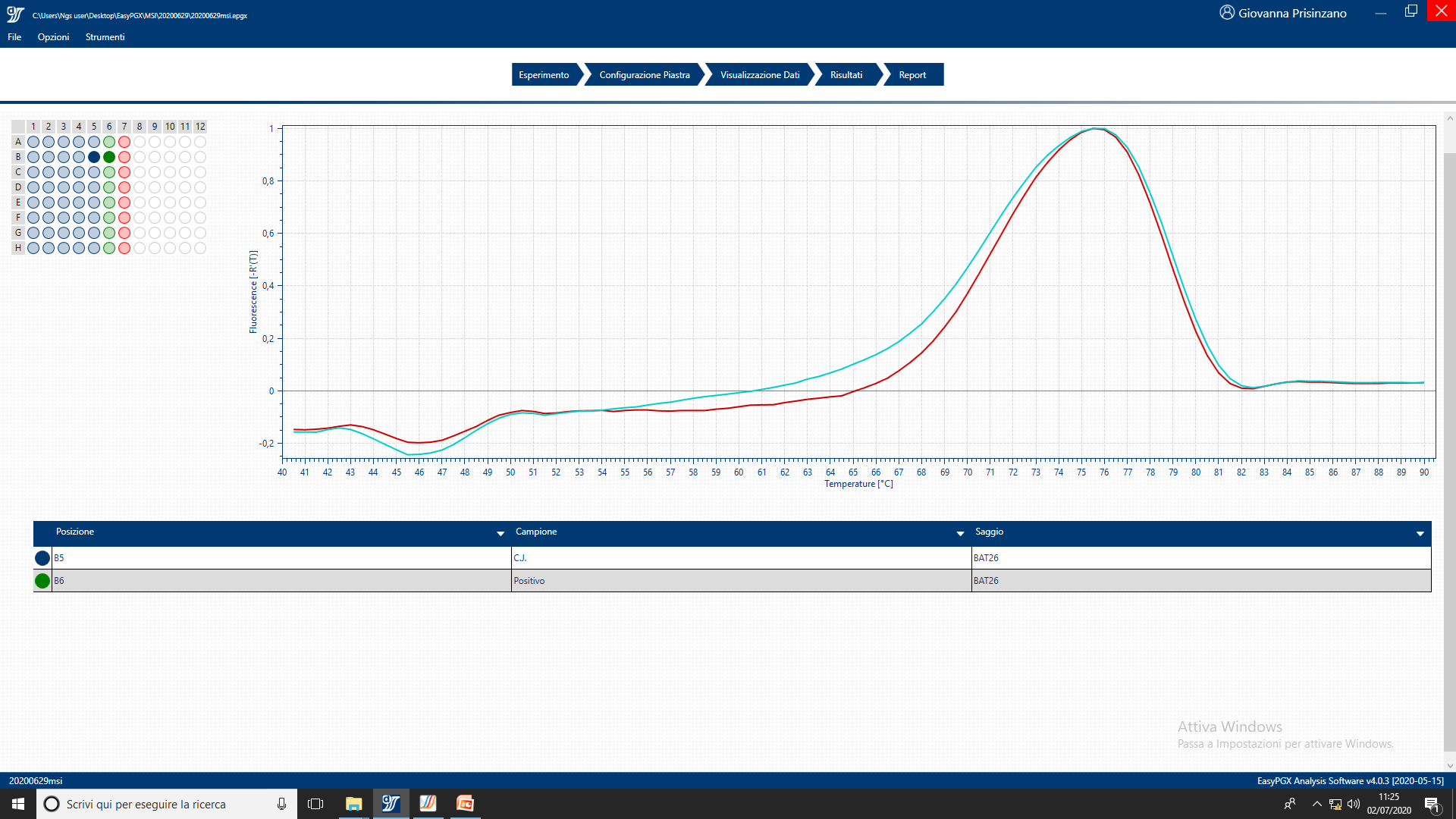


**PASG1**

**C-**


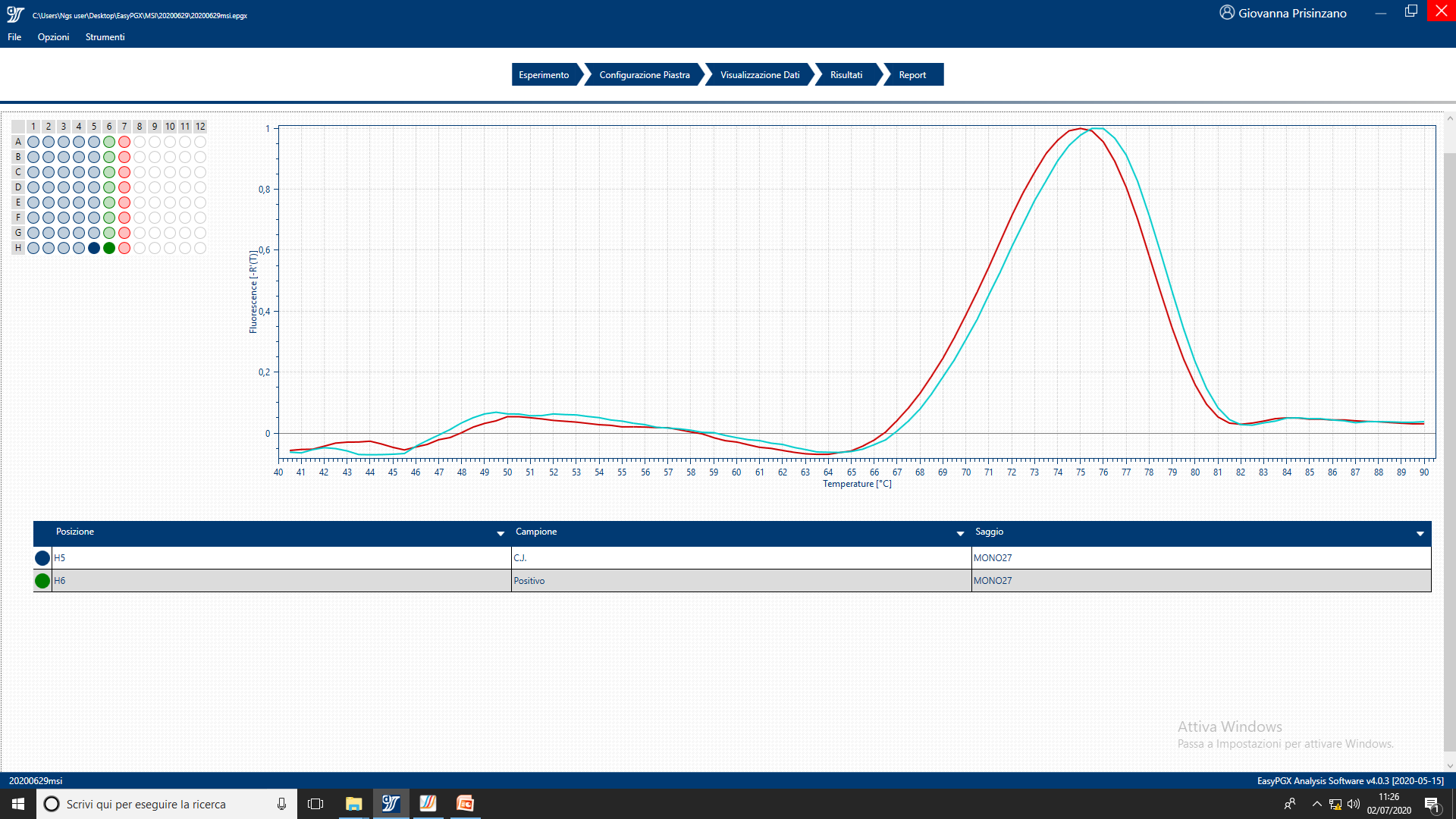

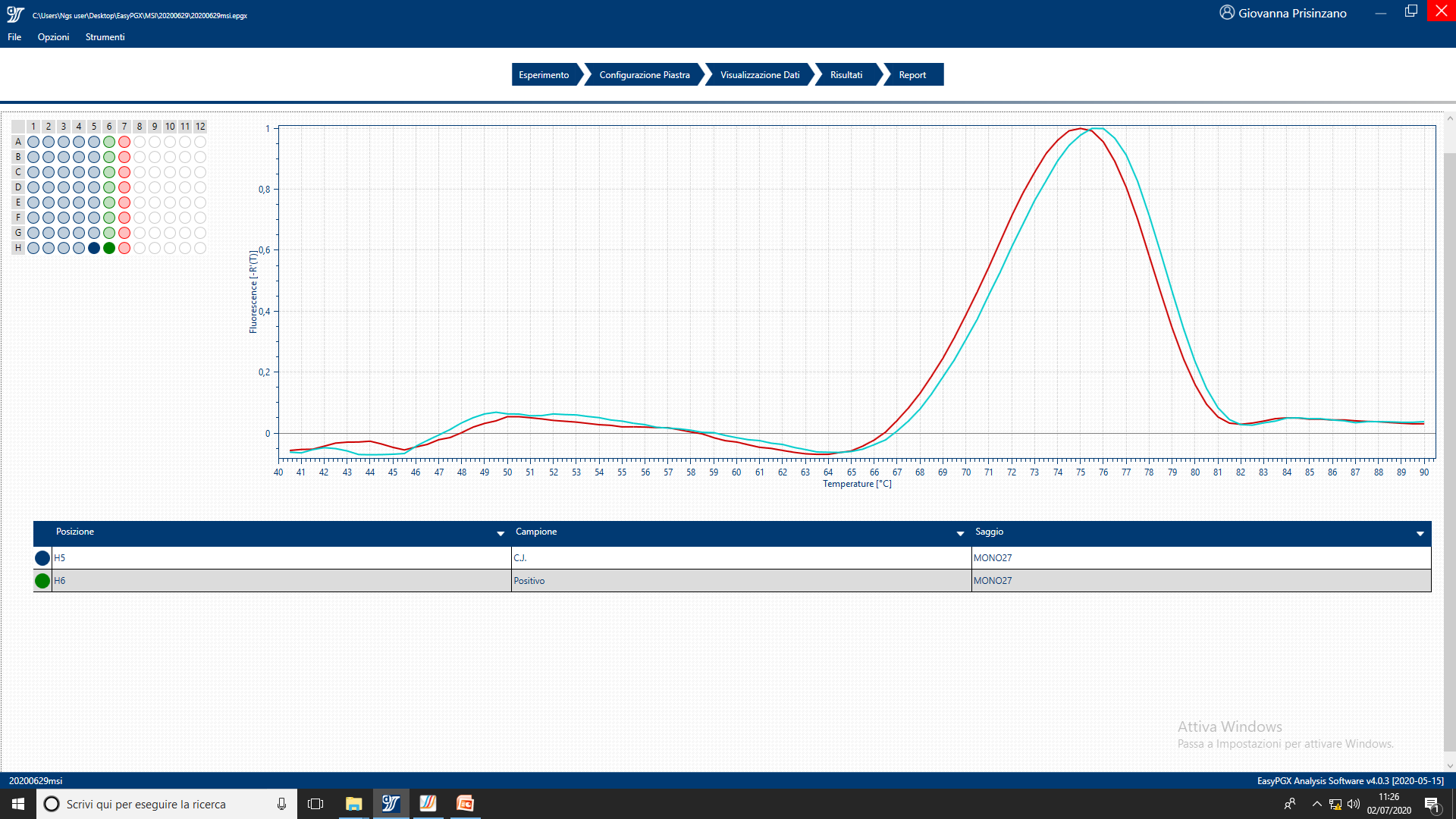


**PASG1**

**C-**


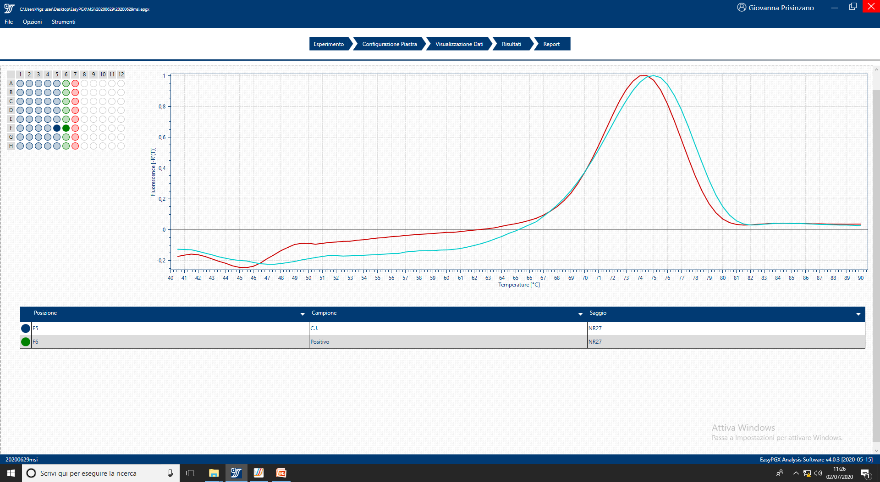

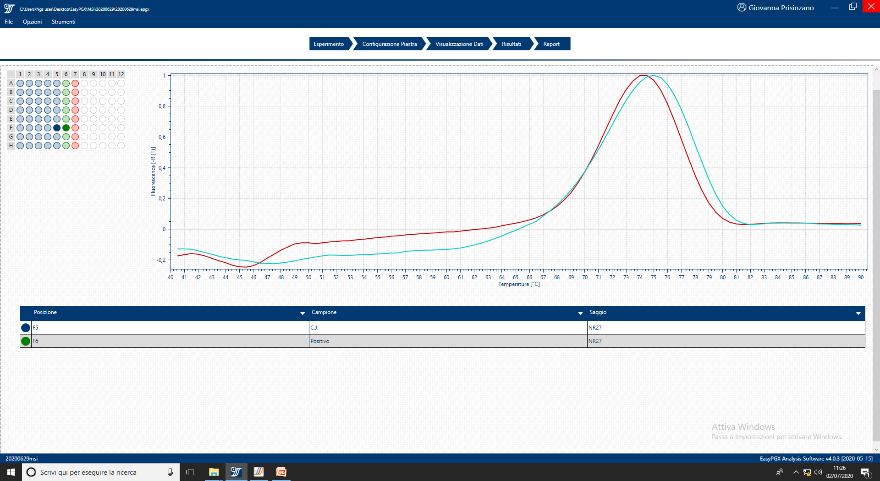


**PASG1**

**C-**


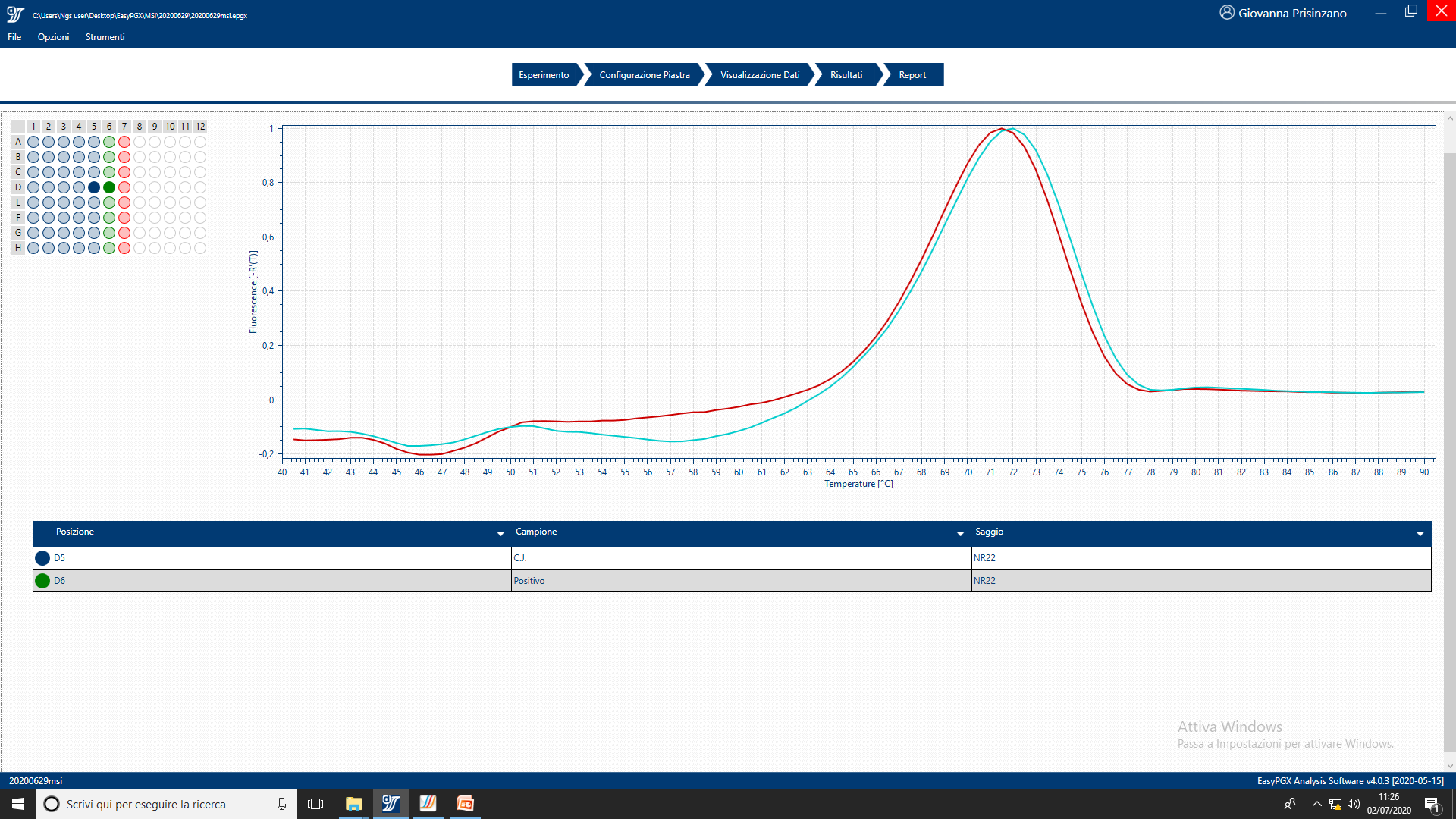

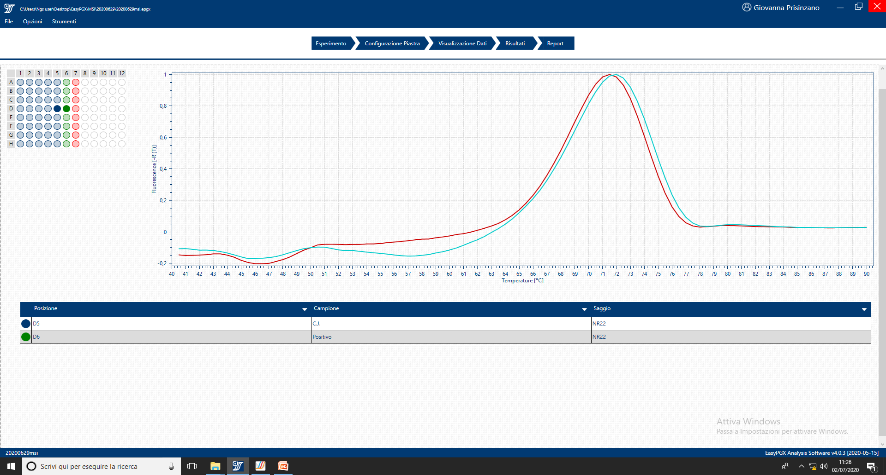


**PASG1**

**C-**


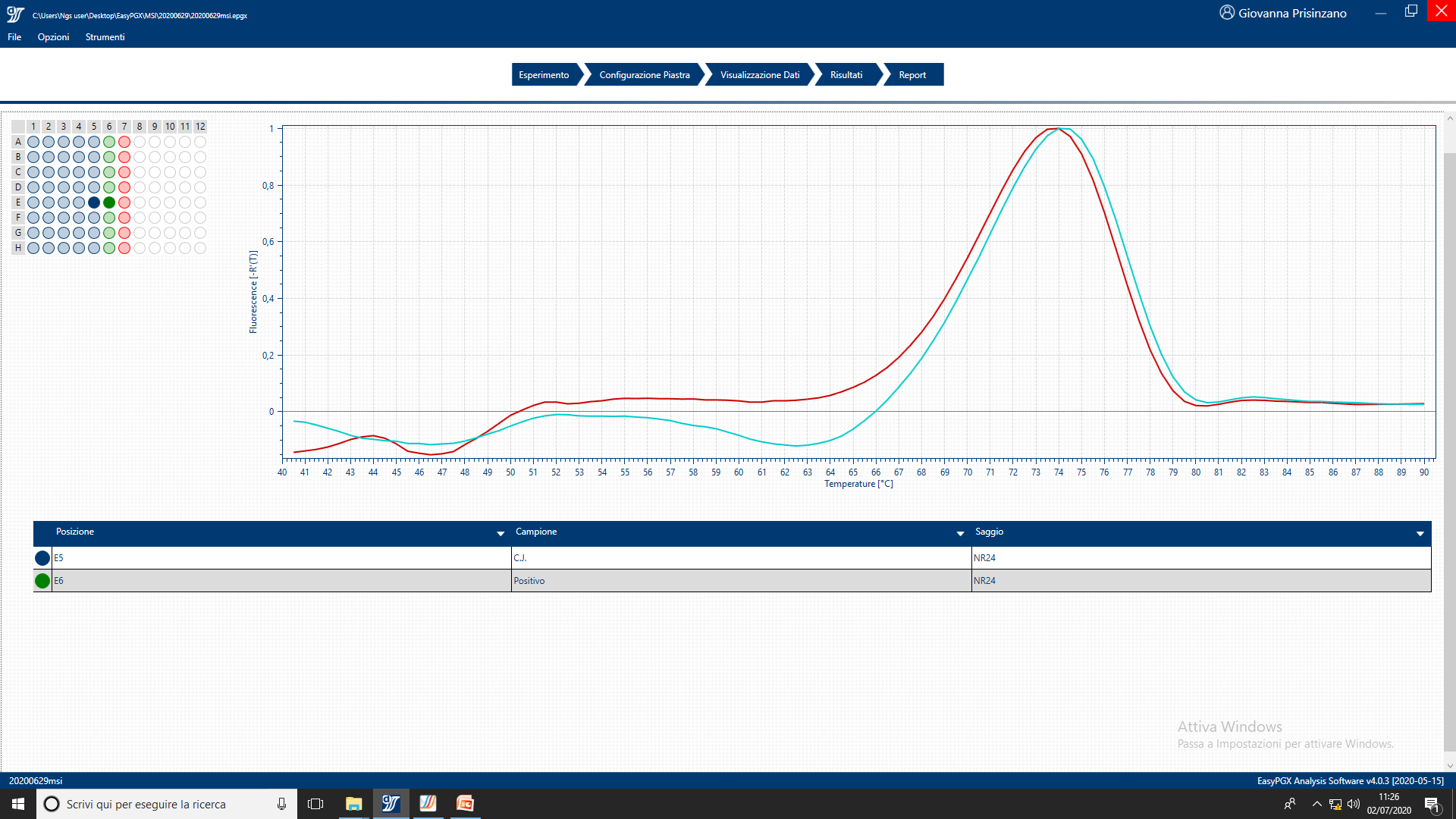

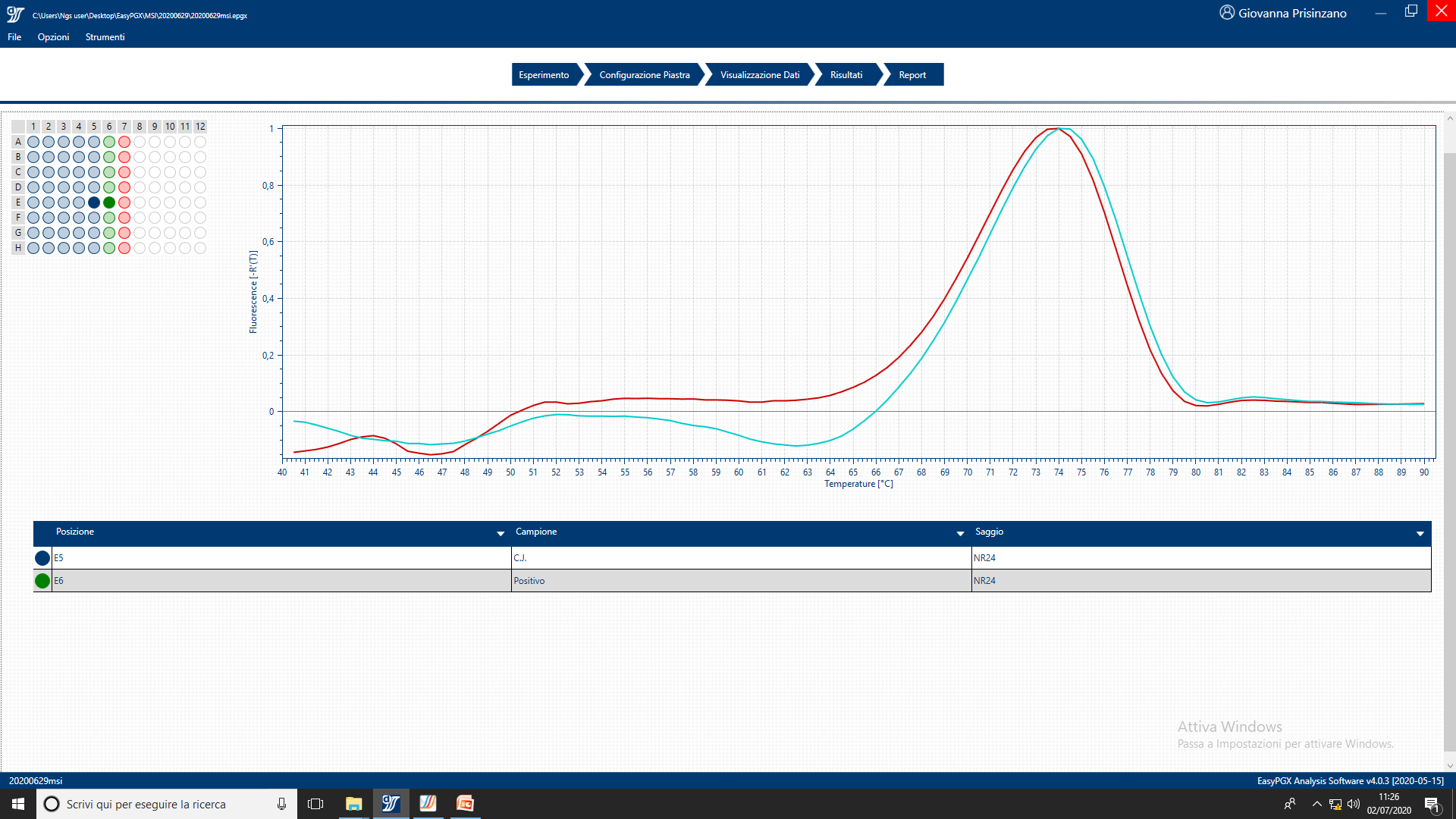


**PASG1**

**C-**


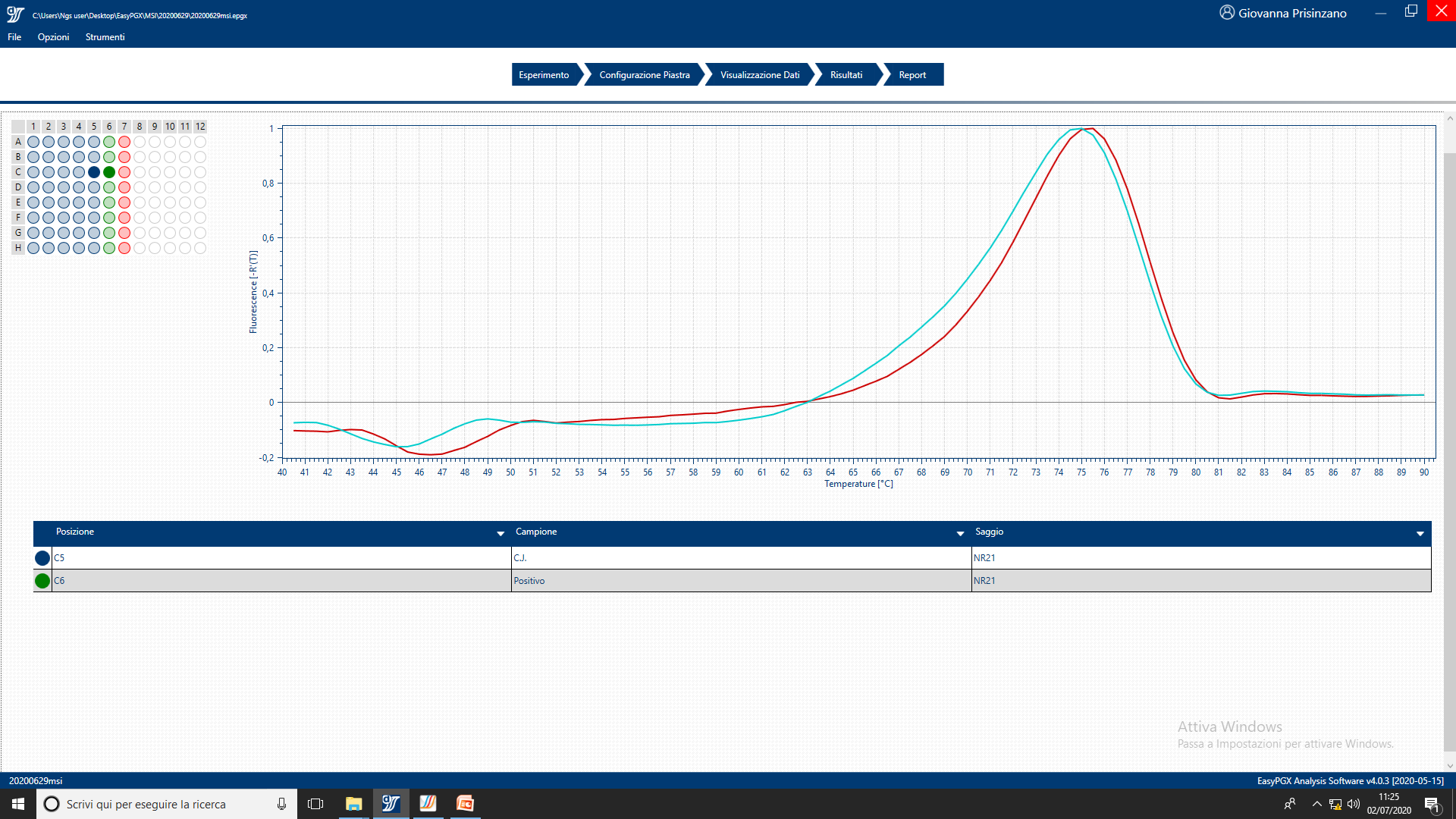

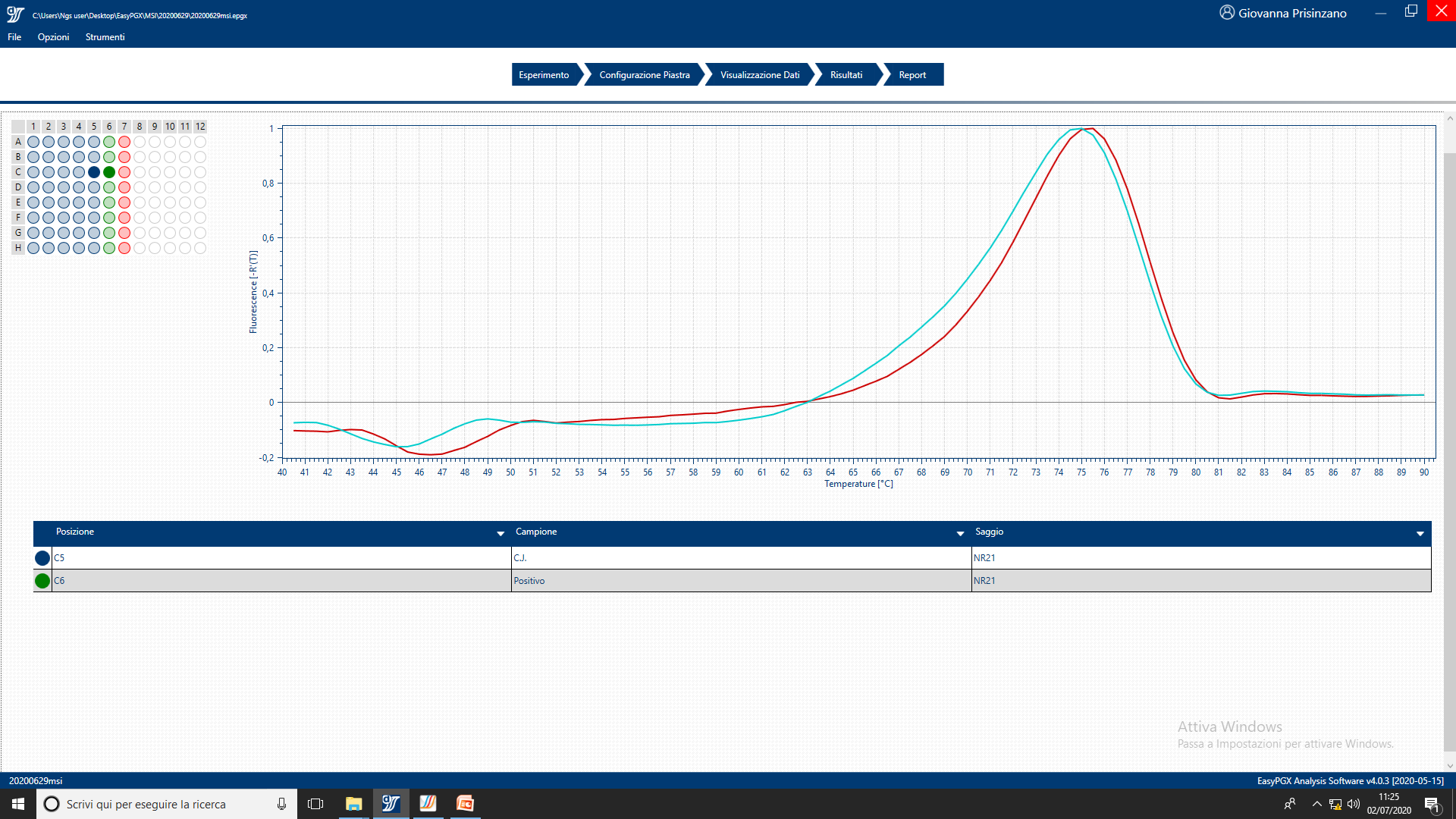


**PASG1**

**C-**


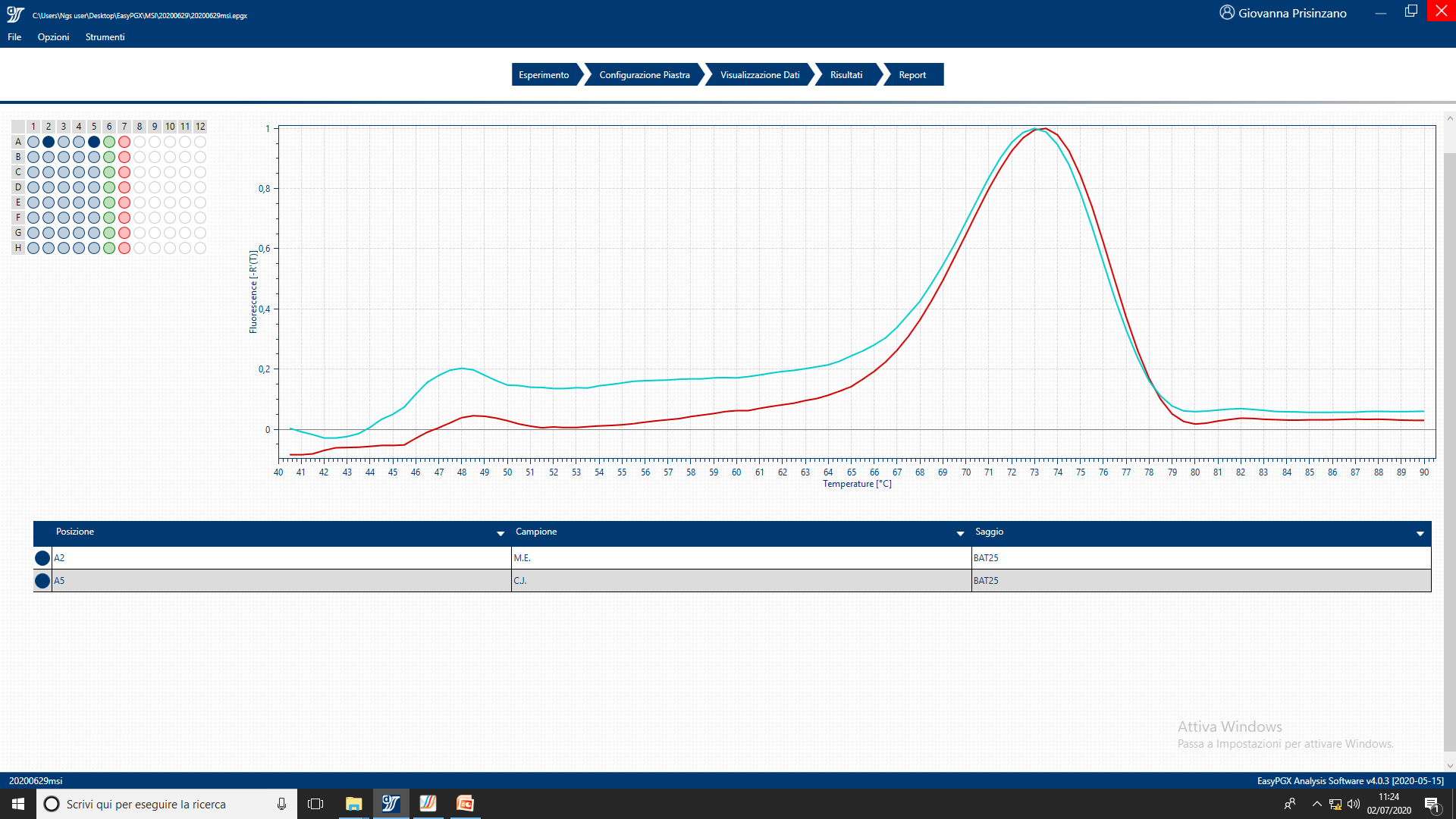

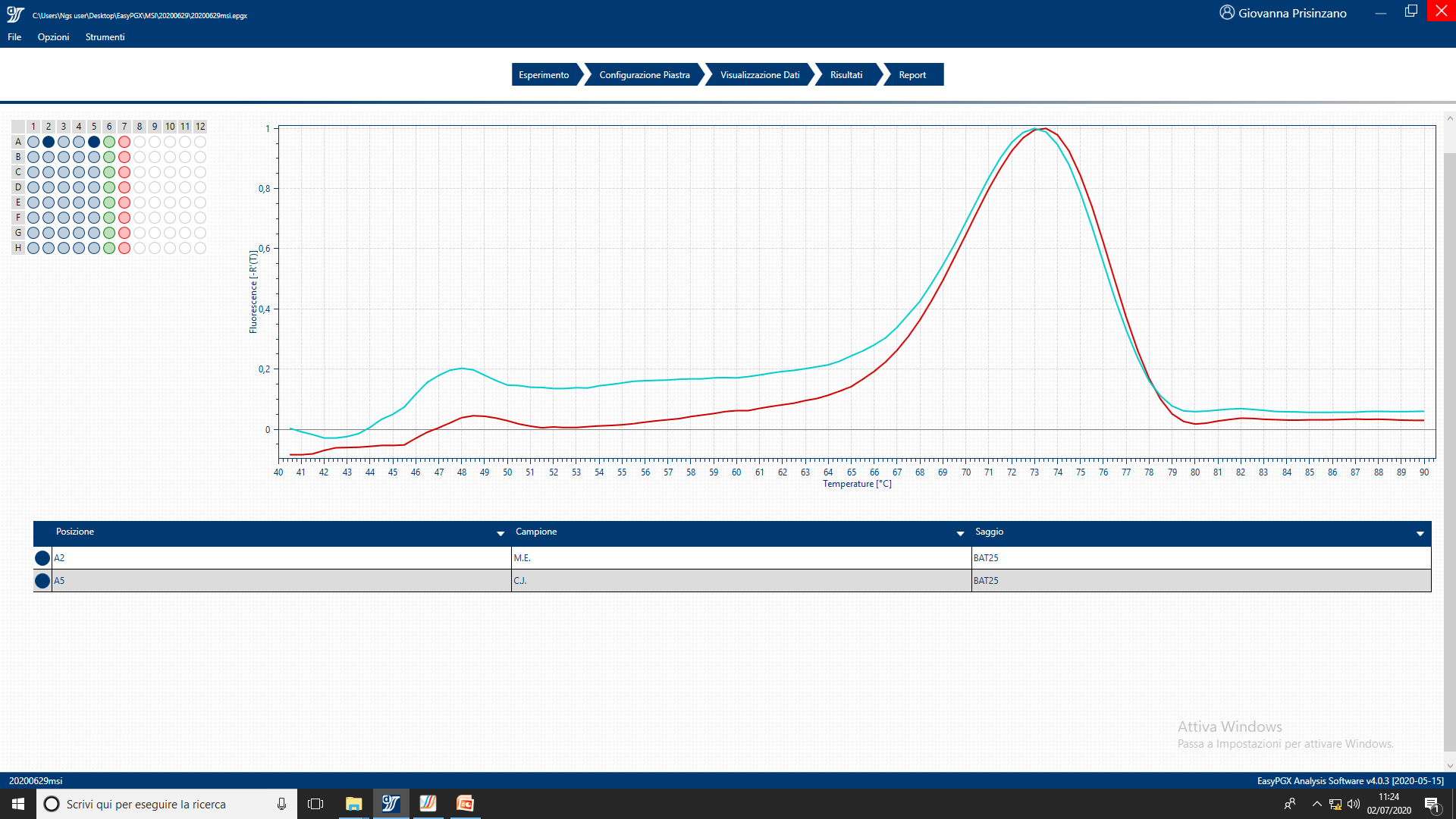


**PASG1**

**C-**

**Supplementary Figure 3.** Representative melt curves of BAT-25, BAT-26, NR-21, NR-22, NR-24, NR-27, CAT-25, MONO-27 in the patient tumor sample (PASG1, blue line) compared to the stable control (C-, red line).
